# Retraction Note: Relationship between bone density and bone metabolism in adolescent idiopathic scoliosis

**DOI:** 10.1186/s13013-015-0057-4

**Published:** 2015-12-03

**Authors:** Ko Ishida, Yoichi Aota, Naoto Mitsugi, Motonori Kono, Takayuki Higashi, Takuya Kawai, Katsutaka Yamada, Takanori Niimura, Kanichiro Kaneko, Hironori Tanabe, Yohei Ito, Tomoyuki Katsuhata, Tomoyuki Saito

**Affiliations:** Department of Orthopaedic Surgery, Yokohama City University Medical Center, Yokohama, Japan; Department of Orthopaedic Surgery, Yokohama City Brain and Stroke Center, Yokohama, Japan; Department of Orthopaedic Surgery, Yokohama City University, Yokohama, Japan

This article [1] has been retracted by the authors and Editor as it was republished by the journal in error. The article was mistakenly resubmitted by the corresponding author as a new manuscript during the production stage of publication, creating a duplicated entry. Unfortunately, this mistake was not detected by the journal and led to duplicate publication of the article [2]. The decision has been made to retract the republished article [1]. The authors and Editor apologize for this error and any inconvenience caused.

The following should be considered the version of record and used for citation purposes: “Ishida K, Aota Y, Mitsugi N, Kono M, Higashi T, Kawai T, Yamada K, Niimura T, Kaneko K, Tanabe H, Ito Y, Katsuhata T, Saito T, Relationship between bone density and bone metabolism in adolescent idiopathic scoliosis, Scoliosis 2015, 10:9, DOI:10.1186/s13013-015-0033-z”.

The duplicate “Ishida K, Aota Y, Mitsugi N, Kono M, Higashi T, Kawai T, Yamada K, Niimura T, Kaneko K, Tanabe H, Ito Y, Katsuhata T, Saito T, Relationship between bone density and bone metabolism in adolescent idiopathic scoliosis, Scoliosis 2015, 10:19, DOI:10.1186/s13013-015-0043-x” is to be ignored.
